# Comparative Metabolite Profiling of Triterpenoid Saponins and Flavonoids in Flower Color Mutations of *Primula veris* L.

**DOI:** 10.3390/ijms18010153

**Published:** 2017-01-13

**Authors:** Lysanne Apel, Dietmar R. Kammerer, Florian C. Stintzing, Otmar Spring

**Affiliations:** 1 Institute of Botany, Hohenheim University, Garbenstr. 30, 70559 Stuttgart, Germany; O.Spring@uni-hohenheim.de; 2 WALA Heilmittel GmbH, Department of Analytical Development & Research, Section Phytochemical Research, Dorfstr. 1, 73087 Bad Boll/Eckwälden, Germany; dietmar.kammerer@wala.de (D.R.K.); florian.stintzing@wala.de (F.C.S.)

**Keywords:** *Primula veris* L., primrose, HPLC-DAD-MS^n^, triterpenoid saponins, flavonoids, anthocyanins, color polymorphism

## Abstract

*Primula veris* L. is an important medicinal plant with documented use for the treatment of gout, headache and migraine reaching back to the Middle Ages. Triterpenoid saponins from roots and flowers are used in up-to-date phytotherapeutic treatment of bronchitis and colds due to their expectorant and secretolytic effects. In addition to the wild type plants with yellow petals, a red variant and an intermediate orange form of *Primula veris* L. have recently been found in a natural habitat. The secondary metabolite profiles of roots, leaves and flowers of these rare variants were investigated and compared with the wild type metabolome. Two flavonoids, six flavonoid glycosides, four novel methylated flavonoid glycosides, five anthocyanins and three triterpenoid saponins were identified in alcoholic extracts from the petals, leaves and roots of the three variants by high performance liquid chromatography (HPLC)-diode array detection (DAD)/mass spectrometry (MS^n^) analyses. Anthocyanins were detected in the petals of the red and orange variety, but not in the wild type. No other effects on the metabolite profiles of the three varieties have been observed. The possibility is discussed that a regulatory step of the anthocyanin biosynthetic pathway may have been affected by mutation thus triggering color polymorphism in the petals.

## 1. Introduction

*Primula veris* L. (Primulaceae), also known under the common names cowslip or cowslip primrose, is native to Europe and Siberia, as well as West and Central Asia. The perennial plant with a short rhizome grows in warm, sunny and dry habitats, especially on dry meadows and light-flooded deciduous forests [1]. Various subspecies and varieties are differentiated, however, the species is also known to hybridize, for instance, with *Primula elatior* L. [2]. Besides the yellow wild type of *P. veris*, a report of a natural orange variety [3] exists, and more recently a red flowered type has been offered commercially in garden plant markets. From a natural habitat in Southern Germany, a population of yellow, red and intermediate orange plants has been known for more than 10 years. This recently offered the possibility to analyze the phytochemistry of these rare varieties, particularly with respect to similarity or differences in pharmacologically interesting compounds known from the wild type.

The metabolome of the wild type of *P. veris* has been well described in the scientific literature. Beside the triterpenoid saponins and carotenoids, essential oils are also present in *P. veris* petals [4], whereby the carotenoids provide the typically yellow flower color. However, phenolic compounds belonging to the flavones and flavonols are the main components of the yellow petals. Already in 1927, the phenolic compound patterns of various *Primula* species have been characterized and used for species identification [5]. Especially for *P. veris*, the flavonoid glycosides quercetin-3-*O*-rutinoside (rutin) as well as kaempferol-dirhamnoside were identified [6]. The discovery of the floral pigments gossypetin and kaempferol-3-*O*-gentiotrioside has also been reported [7]. Furthermore, leucocyanidin, leucodelphinidin and the flavonols myricetin, quercetin, kaempferol and their derivatives have been detected in the leaf extract of the wild type [8,9]. Recent studies have demonstrated the habitats and the respective abiotic factors to influence the flavonoid glycoside pattern in the petals and leaves of *P. veris* [10]. However, only the petals and roots are used pharmaceutically, based on the expectorant and secretolytic effects of the triterpenoid saponins like priverosaponin B-22-acetate, primula acid I and primula acid II [11,12]. Phenolic glycosides, mainly primeverin and primulaverin, are also characteristic compounds of *Primula* roots and have been suggested as indicators of the age of the plant material [12]. Moreover, Mueller et al. [12] described the first liquid chromatographic method suitable for the characterization of bioactive compounds, i.e., saponins and phenolic glycosides, present in *P. veris* and *P. elatior*. A more comprehensive characterization of the metabolite profile with focus on triterpenoid saponins and phenolic compounds (flavonoid glycosides including anthocyanins) in the petals, leaves and roots has not been reported so far. Therefore, we have developed a high performance liquid chromatography-diode array detection/mass spectrometry (HPLC-DAD-MS^n^) method to analyze and compare the secondary metabolite profile of the different plant parts. The study presented here is also focused on the identification of secondary compounds, especially anthocyanins in the *Primula* petals in order to unravel the chemical background of the different *Primula* color variants. Furthermore, the possible correlation between the variations in the flower colors caused by the pigment change and the variation in the metabolite profile of *P. veris* was also investigated. The phytochemical characterization of the *Primula* variants and the different plant shall complement the literature data of the therapeutically used *Primula* species.

## 2. Results

### 2.1. Color Variants of Primula veris

**Figure 1:**
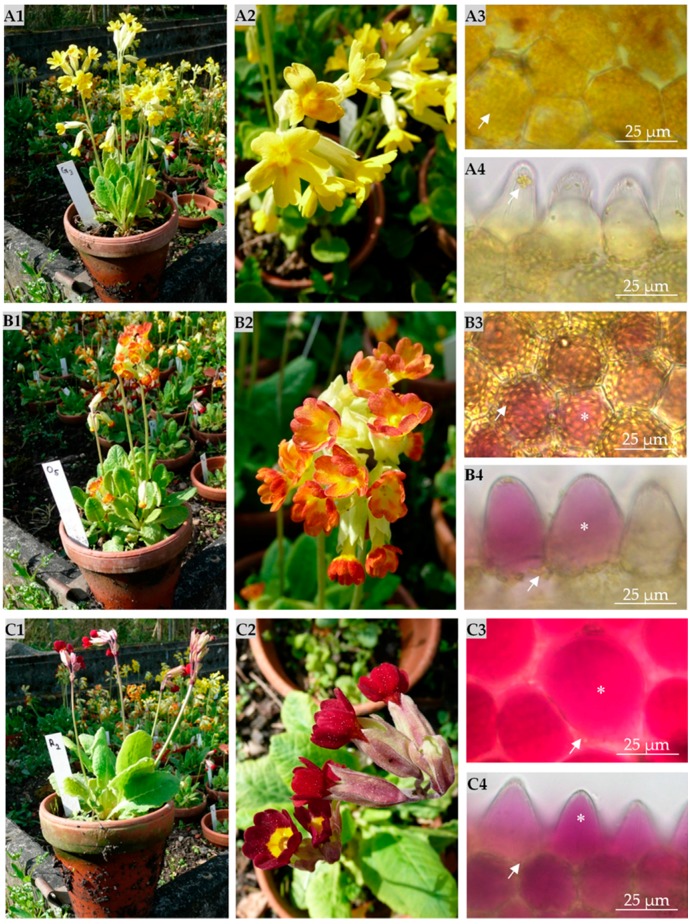
*Primula veris* L. with different petal colors from the botanical garden of Hohenheim University, May 2015. Overview of *P. veris* with yellow petals (**A1**), orange petals (**B1**) and red petals (**C1**). Close-up view of the yellow petals (**A2**), orange petals (**B2**) and red petals (**C2**). Light microscopic images of the differently colored petals in supervision (**A3**,**B3**,**C3**) and in cross-section (**A4**,**B4**,**C4**). Yellow chromoplasts (arrows) are shown in the papilliform epidermis cells of the yellow petals. The vacuoles of the cells are colorless (**A3**,**A4**). The orange colored petals also have yellow chromoplasts. Papillate epidermal cells are characterized by the occurrence of anthocyanins in vacuoles (**B3**,**B4**). Some epidermis cells include anthocyanins (*) in their vacuoles, thus exhibiting red color. All papilliform epidermis cells of the red-colored petals have anthocyanin-containing vacuoles. Yellow chromoplasts (**arrows**) exist, but are masked by the content of the vacuoles (**C3**,**C4**).

Three noticeable color variants of *P. veris* were found in their natural habitat in southern Germany (Figure 1). Besides the typical wild type with yellow petals and orange spots at the base (Figure 1(A1,A2)), a form with deeply red petals around the yellow central part (Figure 1(C1,C2)) occurred. A third variety (Figure 1(B1,B2)) appeared intermediate to the wild type and red type in that the petal color shifted from yellow in the center to red towards the tips, thus imparting the flower an orange appeal. Light microscopy of the different petals revealed the yellow wild type to be devoid of reddish vacuoles, which were found in the epidermal papilla of the orange and red forms, most likely due to the accumulation of anthocyanins (Figure 1(A3,B3,C3–A4,B4,C4)). However, all three variants showed yellow chromoplasts in the epidermal cells colored by carotenoids. This suggested that in the red colored areas, anthocyanins in the papillate cells completely cover the chromoplasts of the basal epidermal cells and that the orange variety represents only an attenuated form, where the yellow pigments are still visible. Besides that, the color varieties shared all other phenotypical features and could not be differentiated from the wild type by distinctive morphological characters. The flower stalks reached 20–25 cm in length harboring 10–30 blossoms. The flowering stage was uniformly reached in April to May.

### 2.2. Metabolite Profiles of the Primula Variants

**Figure 2:**
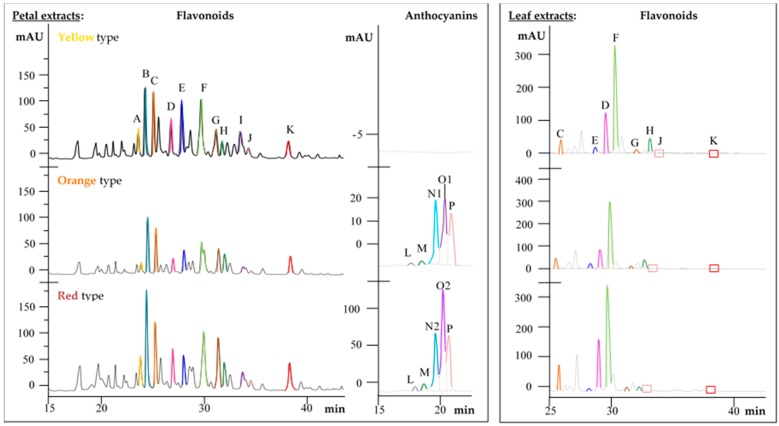
Comparison of high performance liquid chromatograms of *Primula veris* L. petal and leaf extracts covering flavonoid glycosides at 280 nm and anthocyanins at 520 nm. Identical compounds are marked with the same color and the letters A to P: A: (+)-Catechin (yellow); B: Methyl-myricetin-trihexoside (light blue); C: Quercetin-trihexoside (orange); D: Kaempferol-3-*O*-diglucoside-7-*O*-glucoside (pink); E: Quercetin-3-*O*-gentiobioside (blue); F: Kaempferol-3-*O*-galactoside-rhamnoside-7-*O*-rhamnoside (light green); G: Quercetin-methylether-rhamnoside-glucoside-rhamnoside (brown); H: Quercetin-3-*O*-rutinoside (grey); I: Methyl-quercetin-dihexoside (purple); J: Kaempferol-3-*O*-rutinoside (light red); K: Methyl-quercetin-rhamnoside-hexoside (red); L: Cyanidin-hexoside (light purple); M: Cyanidin-glucoside (dark green); N1: Coelution of peonidin-dihexoside and peonidin-hexoside (aqua); N2: Coelution of peonidin-hexoside and malvidin-hexoside (aqua); O1: Coelution of malvidin-hexoside and peonidin-hexoside (purple); O2: Peonidin-dihexoside (purple); P: Malvidin-hexoside (apricot).

HPLC-DAD-MS^n^ analyses of petal extracts of all three Primula varieties showed a broad spectrum of flavonoids and their glycosylated or methylated derivatives (Figure 2). By means of mass spectrometric data and database comparison, the following known compounds were assigned for all *Primula* variants: (+)-catechin, kaempferol-3-*O*-galactoside-rhamnoside-7-*O*-rhamnoside, kaempferol-3-*O*-rutinoside, kaempferol-3-*O*-diglucoside-7-*O*-glucoside, quercetin-3-*O*-rutinoside, quercetin-3-*O*-gentiobioside, quercetin-trihexoside and primula acid I. Four methylated flavonoid derivatives tentatively assigned to methyl-quercetin-dihexoside, methyl-quercetin-rhamnoside-hexoside, quercetin-methylether-rhamnoside-glucoside-rhamnoside and methyl-myricetin-trihexoside were found for the first time and characterized according to mass spectrometric data reported in the literature. The only difference between the three Primula samples was found in isorhamnetin (data not shown), which occurred in very small amounts in the orange and red varieties, but was not detected in the yellow wild type petals.

In addition, the petal extracts contained the saponin primula acid I in all three varieties. The metabolic difference between the color varieties was found in the HPLC patterns of methanol-water extracts of the petals, which allowed the determination of anthocyanins (Figure 2, central chromatograms). While the orange and red variety both contained cyanidin-hexoside, cyanidin-3-*O*-glucoside, peonidin-hexoside, peonidin-dihexoside and malvidin-hexoside, these compounds were not detected in the yellow wild type plants. All other identified flavonoids, flavonoid glycosides and triterpenoid saponins were uniformly found in the petals of all three color variants with only marginal quantitative deviations (Figure 2, left chromatograms).

Leaf extracts of the three *Primula* samples were characterized by very similar flavonoid and saponin profiles compared to the petals; however, individual compounds were present in different relative quantities (Figure 2, right chromatograms). Kaempferol-3-*O*-galactoside-rhamnoside-7-*O*-rhamnoside (peak F in Figure 2) was the most prominent compound in all three varieties. In the red colored type, kaempferol-3-*O*-diglucoside-7-*O*-glucoside and quercetin-trihexoside appeared slightly increased in their contents when compared with the wild type, and the orange variety contained somewhat smaller amounts of all compounds. In those parts of the chromatograms exhibiting more lipophilic compounds, trace amounts of kaempferol-3-*O*-rutinoside (light red box J) and methyl-quercetin-rahmnoside-hexoside (red box K) were detected and their occurrence verified by mass spectrometry (MS) data.

The root extracts of all three *Primula* samples provided identical compound profiles, which consisted of triterpenoid saponins, but were devoid of the flavonoids and anthocyanins detected in the above ground plant organs (Table 1). Besides, primula acid I, primula acid II and priverosaponin B-22-acetate were found in roots, thus supporting previous findings for *P. veris* [12,13,14]. All identified compounds with their characteristic spectrometric data and their occurrence in petals, leaves and roots of the three *Primula* variants are summarized in Table 1.

### 2.3. Comparison of Metabolite Profiles between Plant Parts

The characterization of different plant parts with regard to their secondary metabolite profile revealed that eight out of ten flavonoid glycosides identified in the petals were also detected in the leaves. Of the methylated flavonoids, only quercetin-methylether-rhamnoside-glucoside-rhamnoside and methyl-quercetin-rhamnoside-hexoside were detected in leaves, whereas methyl-quercetin-dihexoside and methyl-myricetin-trihexoside were missing or below the limit of detection. A range of yet unknown peaks in a retention time range of 50 to 60 min were detected only in leaf extracts but not in petal extracts, however, the yields of these components were insufficient for structure identification (data not shown). Additionally, the main triterpenoid saponin primula acid I occurred in each of the different plant parts. In contrast, primula acid II and priverosaponin B-22-acetate were exclusively detected in *Primula* roots (Table 1).

### 2.4. Identification of Novel Compounds in the Primula Variants

Three novel methylated quercetin derivatives were detected in the petals or leaf extracts for the first time. The fragment ions at *m*/*z* 315 and 300 were important for their identification and revealed the presence of an aromatic methoxyl group. Tentative structure assignment was performed as follows. Methyl-quercetin-dihexoside was identified in petal extracts from a peak with a retention time of 33.2 min by means of mass spectra revealing a characteristic [M-H]^−^ ion at *m*/*z* 639 and fragment ions at *m*/*z* 477 and 315 ([M-H162162]^−^). Methyl-quercetin-rhamnoside-hexoside was detected in petals as well as leaves. The fragmentation in the MS^2^ experiment revealed the loss of a rhamnose moiety ([M-H146]^−^) with a fragment ion at *m*/*z* 477, followed by an *O*-hexose cleavage ([M-H-146-162]^−^) resulting in the corresponding fragment ion at *m*/*z* 315. Due to the lack of literature data and insufficient sample amounts for NMR investigations, further characterization of the two methyl-quercetin derivatives was not performed. Based on the study of Pemp and Krenn [13], the quercetin-methylether-rhamnoside-glucoside-rhamnoside could be characterized in more detail. Compound assignment was based on its UV spectrum (maxima at 254 nm and 354 nm) as well as mass spectrometric data, which revealed a base peak at *m*/*z* 769 and a fragment ion at *m*/*z* 737 indicating methylether cleavage by releasing methanol. The loss of rhamnose [M-H-146]^−^ and glucose [M-H-162]^−^ moieties were found in further fragmentation steps.

Another flavonoid glycoside, which has not been described in *P. veris* before, was detected in petals. MS data of the compound with a retention time of 24.3 min suggested the occurrence of a methyl-myricetin-trihexoside with characteristic fragment ions at *m*/*z* 817, 655 and 493. The MS^2^ experiments revealed the losses of three *O*-hexose moieties. The assignment of the flavonol aglycone myricetin was based on identical MS data reported by Kim et al. [14].

The anthocyanins, exclusively found in orange and red *Primula* petals, were also assigned based on UV and mass spectrometric measurements. Cyanidin-3-*O*-glucoside showed identical HPLC behavior (retention time 18.1 min) and spectroscopic characteristics (UV_max_ 280 and 520 nm) compared to the corresponding reference standard. The fragment ions at *m*/*z* 449 and 287 ([M-162]^+^) reported in the literature were confirmed. A second cyanidin-hexoside was detected exhibiting a retention time of 17.3 min. Additionally, peonidin and malvidin derivatives were identified and confirmed by comparison with literature data of Wu et al. [19]. Peaks N1,2 and O1,2 (Figure 2, central chromatograms) appeared to be coelutions of several compounds, which could only partly be resolved and indicated most likely quantitative differences between the orange and red color variants. A mixture of peonidin-dihexoside and peonidin-hexoside (*m*/*z* 625 and 463, respectively) and the typical fragment ion at *m*/*z* 301 for the peonidin aglycone at a retention time of 19.3 min was detected in the extract of the orange petals. Peak N2 in the red variety contained a substance mixture of malvidin-hexoside, peonidin-dihexoside and peonidin-hexoside. Malvidin-hexoside exhibited absorption maxima at 260 and 520 nm and a molecular ion [M]^+^ at *m*/*z* 493 as well as a fragment ion at *m*/*z* 331, thus indicating the loss of a hexose moiety. Furthermore, the peak at a retention time of 20.0 min revealed the presence of malvidin-hexoside and peonidin-hexoside in the orange petal extract. The peak with the same retention time in the red petal extract revealed only the occurrence of peonidin-hexoside. Finally, malvidin-hexoside was re-assigned to the peak with a retention time of 20.5 min in both orange and red petals. A tailing or fronting might be the reason for this multiple assignment of probably the same peaks. Consequently, this coelution aggravated unambiguous compound assignment, which is assumed to be as follows: N: Peonidin-dihexoside, O: Peonidin-hexoside and P: Malvidin-hexoside. All identified substances with their characteristic spectrometric data and their occurrence in the petals, leaves and roots of the three *Primula* variants are summarized in Table 1.

## 3. Discussion

Within the past century phytochemical studies of 100 species from 25 different genera of Primulaceae have been conducted [7]. Flavonoids and saponins were among the predominant compounds characteristic for this family. However, significant diversity was found with respect to specific compounds or derivatives and in their occurrence in different plant organs. This is particularly relevant for the pharmacological use of *Primula* species, among which *P. veris* plays the most important role.

Previous studies reported the petal pigment kaempferol-3-gentiotrioside from *P. veris* and 39 further *Primula* species [7]. Furthermore, the leaf pigment quercetin-3-*O*-rutinoside was also found in *P. veris* before [7]. The occurrence of these two flavonoid glycosides was confirmed in the present work, and both were found in petals as well as leaves. Further quercetin derivatives have already been identified in previous studies of *P. veris* and *P. elatior* and were used for analytical differentiation between these two medicinal plants [20]. Quercetin-3-gentiobioside, quercetin-3-*O*-gentiotrioside, kaempferol-3-*O*-rutinoside and kaempferol-3-*O*-robinobioside were isolated from petals of the wild type *P. veris* [7] and were also detected in flowers and leaves of the current study.

In addition, we were able to identify three methylated quercetin-derivatives and methyl-myricetin-trihexoside in the petal extracts, which, to the best of our knowledge, have not yet been reported in the species before. Mono-, di-, tri- and penta-methoxyflavones have been identified and characterized during the last years by liquid chromatography and mass spectrometry in flowers of *P. veris* and have long been recognized to possess antiallergic, antiviral, anti-inflammatory activities. They also play an important part in the biochemistry and physiology of plants, for example they act as antioxidants, enzyme inhibitors and precursors of toxic substances [21,22,23,24]. However, glycosylated methoxyflavones for *P. veris* are unknown [23]. The exact structural identification of the novel methylated quercetin- and myricetin-derivatives was not possible due to lack of sufficient amounts required for extensive 2D-NMR experiments. However, the structural elements identified from the MS/MS measurements suggested similarity to compounds recently described from phytochemical studies of other plant species known for pharmacological use. Thus, the methyl-quercetin-rhamnoside-hexoside from the petals and leaves of the yellow, orange and red variants resembles 4′-O-methylquercetin-3-rutinoside recently extracted from *Albizzia amara* leaves [25]. The methyl-quercetin-dihexoside with the molecular weight of 640 displayed characteristics of tamarixetin-3,7-diglucoside, a compound found in the aerial parts of *Echium longifolium* and *Heliotropium digynum* [26]. A compound structurally similar to the methyl-myricetin-trihexoside from *P. veris* was found in *Artemisia annua* [27].

In contrast to the flavonoids quercetin and kaempferol, myricetin occurs very rarely in the family Primulaceae [7] and has not been found in *P. veris* so far [28]. Among the flavonoid aglyca, (+)-catechin was also identified for the first time in the petals of this species. Isorhamnetin was found only in trace amounts in petal extracts of the orange and red variants. The absence of this compound in the wild type is most likely attributed to the extremely low compound concentration. This assumption is supported by the fact that Karl et al. [20] have previously reported the extraction of isorhamnetin and several of its glycosylated derivatives from the petals of *Primula officinalis* Hill. (a synonym for *P. veris* L.). Strong variation in the compound concentration may also account for the absence of the flavonoids gossypetin, luteolin and apigenin in our samples, which have been reported for *P. veris* before [20].

Triterpenoid saponins are among the pharmacologically most interesting compounds of *Primula* species. Primula acid I was detected as major triterpenoid saponin in all three plant organs, thus confirming former reports where the compound was isolated from *P. veris*, *P. elatior* and *Primula vulgaris* [29]. The saponins primula acid II and priverosaponin B-22-acetate, which were exclusively found in *P. veris* roots, are also secondary metabolites of *P. elatior*. According to literature data, primeverin is the main glycoside of *P. veris* roots [12]. Interestingly, we were not able to detect primeverin in the roots of any of the three *P. veris* variants considered in this study.

The substance identification of the anthocyanins is unambiguous. Extracts of the yellow petals were devoid of anthocyanins. This coincided with the lack of red colored vacuoles in the papillate epidermal cells visualized by light microscopy. For the wild type, this result is confirmed by earlier studies, where *P. veris*, *P. vulgaris* and *P. elatior* were characterized by the lack of anthocyanins, whereas carotenoids were detected in all three *Primula* species [7]. The orange and red *Primula* types analyzed in our study contained cyanidin-3-*O*-glucoside, cyanidin-hexoside, peonidin-hexoside, peonidin-dihexoside and malvidin-hexoside. These water-soluble vacuolar pigments have not been described in *P. veris* before. However, anthocyanins like malvidin glycosides and their derivatives are very widespread in some other European *Primula* species [7] and were also detected in the Japanese ornamental plant *Primula sieboldii* [30]. Peonidin, delphinidin, cyanidin and rosinidin glycosides occur occasionally [7].

In conclusion, the comparison of the plant secondary metabolites demonstrated that the three *P. veris* color types only differ in the occurrence and profile of the petal anthocyanins. Other compounds detected in the present study were not found to be affected by the mutation. This accounts particularly for saponins, which, due to their expectorant and secretolytic effects, are most relevant for the pharmacological use of *P. veris*. Currently, only the roots and petals of the wild type are used as a mild expectorant for the treatment of coughs and bronchitis; however, our study demonstrates that saponins are also present in the leaves. This confirms earlier studies, which also reported the extraction of saponins from *Primula* leaves [11], whereas their concentrations are lower in leaves as compared to petals [12].

The color polymorphism of *P. veris* is still not completely understood, but also known in *P. vulgaris* with petal colors ranging from white, pink and red to violet [3]. In Western Europe, *P. vulgaris* shows a uniform appearance with pale-yellow petals. However, in Eastern Europe (Caucasus, Greece, Turkey and Iran), common primrose is known with pale-yellow, pink, violet and purple petals side by side in one population. Morphological investigations of plants found along the northeast coast of the Black Sea (250 km) showed that the petal color is the only phenotypic difference between the varieties. A classification into different subtypes was therefore found unnecessary [31]. Recent molecular analyses based on chloroplast DNA and internal transcribed spacer markers demonstrated the color polymorphism not to be related to general phylogenetical trends in the population of *P. vulgaris*. Rather, the color polymorphism of *P. vulgaris* was suggested to reflect the postglacial distribution from different refugia and is assumed to be originated by the cross territorial distribution [32]. Molecular investigations aiming at the explanation of color polymorphism of *P. veris* are still pending. Genetic modifications of the anthocyanin biosynthetic pathway may be one option for the occurrence of color variations, whereby a change in gene regulation of the pathway is more likely than a mutation in one of the biosynthetically active enzymes. The ability to synthesize anthocyanins is most likely also present in the yellow wild type, but is not activated in the yellow parts of wild type petals. Observations in the progeny of our plants indicate an intermediate inheritance of the red color phenotype (data not shown). Mutations in the regulation of flavanone-3β-hydroxylase (F3H), a key enzyme of the flavonoid biosynthetic pathway, have the consequence that neither flavonols nor anthocyanins are synthesized, as it was demonstrated for *Ipomoea purpurea* (Convolvulaceae) with a modified pale-yellow phenotype [33]. In the case of *P. veris*, quercetin- as well as kaempferol-derivatives are synthesized. Consequently, mutation in the regulation downstream of F3H, especially the dihydroflavonol 4-reductase (DFR), or in transcription regulator gene(s) were suggested to be reasons for color polymorphism and result in modified phenotypes [34].

Differences in the gene regulation of *P. veris* are already known to be responsible for variation in the development of reproductive structures. *Primula* plants produce two morphological types of flowers: the long-styled flower type forms anthers attached midway along the floral tube (L-morph) and the short-styled flower forms anthers at the top of the floral tubes (S-morph). The flower phenotype is genetically associated with heterostyly genes, a system of heteromorphic self-incompatibility [35]. Recent studies identified 113 candidate heterostyly genes that revealed significant morph-specific differential expression in *P. veris*. One of these candidate genes has been duplicated in *P. veris* and is completely silenced in the long-styled flower type [36]. Similarly, a mutation in a regulatory step of the anthocyanin biosynthetic pathway might be the trigger of color polymorphism in petals.

## 4. Materials and Methods

### 4.1. Plant Material

*Primula veris* L. with different petal color variants from a natural habitat (Vaihingen Enz, Baden-Wuerttemberg/Germany) were cultivated in the Botanical Garden of Hohenheim University. Plant samples were harvested at flowering stage in April and May. Prior to analysis, petals, leaves and roots were manually separated and kept at −20 °C. Specimens were deposited at the herbarium of Hohenheim University (HOH-014883 to HOH-014985).

### 4.2. Chemicals

Reference standards quercetin-3-*O*-rutinoside (rutin), (+)-catechin, luteolin, quercetin and apigenin, kaempferol, isorhamnetin and cyanidin-3-*O*-glucoside were obtained from Carl Roth GmbH (Karlsruhe, Germany). Primeverin and primula acid I were purchased from Phytolab GmbH (Vestenbergsgreuth, Germany).

### 4.3. Sample Preparation

The sample composition of the plant parts composes 1 g of four individuals of the same *Primula* type. Petals, leaves and roots (4 g fresh weight) of the different *P. veris* types were cut into small pieces and extracted with 40 mL of methanol at room temperature for 30 min. The extraction with methanol was repeated and intensified by 30 min incubation in an ultrasonic bath (VWR, Darmstadt, Germany) at 22 °C. Subsequently, the extracts were decanted, filtered, transferred into a round-bottomed flask and kept at 8 °C until HPLC analysis. Petal extracts were also used for anthocyanin analyses. For this purpose, the manually separated petals were extracted with chloroform at room temperature for 30 min plus 30 min under ultrasonication to remove carotenoids from the plant material. The supernatant was removed, subsequently, petals were extracted with 40 mL of methanol/water (8:2 *v*/*v*, pH 3) as described above. All sample preparations were performed in duplicate.

### 4.4. Microscopic Investigations

Fresh petals of the different *Primula* types were investigated with an Axioplan light microscope (Zeiss, Göttingen, Germany) coupled to a digital camera (Canon Deutschland GmbH, Krefeld, Germany). Petals were fixed in small polystyrene blocks and cut in cross and longitudinal sections.

### 4.5. HPLC-DAD Analysis

For chromatographic separation, a Thermo Fisher Scientific Dionex Ultimate 3000 RSLC system (Thermo Fisher Scientific GmbH, Dreieich, Germany) equipped with a vacuum degasser, a binary pump, an autosampler, a thermostatic column compartment (25 °C) and a diode array detector (DAD) was used. Gradient binary eluent system (eluent A: 0.1% formic acid (*v*/*v*); eluent B: acetonitrile) was applied using a SunFire RP C18 column (150 mm × 2.1 mm, Waters, Wexford, Ireland) at a flow rate of 0.21 mL/min with the following gradient: 0–2 min, 0% B; 2–4 min, 0%–5% B; 4–10 min, 5%–10% B; 10–14 min, 10%–15% B; 14–33 min, 15%–20% B; 33–41 min, 20%–35% B; 41–54 min, 35%–100% B; 54–58 min, 100% B; 58–64 min, 100%–0% B; 64–66 min, 0% B. The detection wavelengths were set at 210, 254, 280, 366 and 520 nm. For HPLC-DAD analyses the samples were diluted with purified water (1:1, *v*/*v*). Injection volume was 20 µL (equivalent to 10 mg fresh weight). Chromeleon software (Version 7.2, Dionex, Idstein, Germany) was used for data acquisition and processing.

### 4.6. LC-MS^n^ Analyses

Mass spectrometric analyses were performed using an Agilent 1200 HPLC system (Agilent, Waldbronn, Germany) connected to an HCT ultra ion trap MS detector interfaced with an ESI ion source (Bruker Daltonik, Bremen, Germany). Negative ionization mode was used for phenolic compound and triterpenoid saponin analysis, whereas anthocyanins were analyzed in the positive ionization mode. The following device parameters were applied: dry gas flow (N_2_), 8 L/min; nebulizer pressure, 40 psi; capillary temperature, 350 °C; for the negative and positive ionization modes, MS spectra were recorded in a range of *m*/*z* 50 to 1500 with a compound stability and trap drive level of 100%. The software Agilent Chemstation (Rev. B.01.03 SR1) (Agilent, Waldbronn, Germany) and Bruker Daltonik esquire control (Version 6.1) (Bruker Daltonik GmbH, Bremen, Germany) were used for data acquisition.

### 4.7. Characterization of Phenolic Compounds and Triterpenoid Saponins

The identification of compounds previously described in the literature was realized by comparison of retention times, mass spectrometric and UV/Vis data with those of literature reports and reference compounds, respectively.

The flavones (+)-catechin and isorhamnetin were identified by comparison with the corresponding reference standards. However, isorhamnetin was detected only in trace amounts in the orange and red petal extracts, and was not detected in the yellow wild type petal extract and the corresponding leaf extracts as well as root extracts of the three *Primula* types. The flavonoids apigenin and luteolin were not detected in any of the plant parts of the three *Primula* types, which is in accordance with previous reports. Kaempferol-3-*O*-galactoside-rhamnoside-7-*O*-rhamnoside (*m*/*z* 739) was assigned to the peaks with retention times of 29.5 min (petal extracts) and 30.4 min (leaf extracts) and was identified based on MS^n^ spectra with the base peak at *m*/*z* 739 as well as comparison with literature data [1]. This flavonoid glycoside revealed fragment ions at *m*/*z* 593, 429 and 285, thus indicating the losses of rhamnoside and hexoside moieties and a dehydration reaction. The characteristic fragments of kaempferol (*m*/*z* 257, 241, 229, 213, 199, 151) were confirmed by literature data [18]. Kaempferol-3-*O*-rutinoside was also detected in *Primula* leaves as well as petals and showed MS base peaks at *m*/*z* 593. Fragment ions at *m*/*z* 447 and 285 demonstrated the release of hexose and rhamnose moieties. A comparison with literature data of Sánchez-Rabaneda et al. [15] confirmed the compound identification. Kaempferol-3-*O*-diglucoside-7-*O*-glucoside showed an [M-H]^−^ ion at *m*/*z* 771 and the release of three hexose moieties, thus yielding characteristic fragment ions at *m*/*z* 609, 447 and 285 in the MS^2^ experiments. These data are in agreement with the findings of Gonzales et al. [17] and Harborne [7]. The flavonol quercetin and its glycosides were identified in *Primula* petals and leaves. For the identification of the respective aglycone quercetin, MS^n^ spectra of Fabre and Rustan [16] were used, which confirmed the main ion at *m*/*z* 301 and the characteristic fragment ions at *m*/*z* 273 and 257 indicating the losses of CO as well as CO_2_. Fragment ions at *m*/*z* 193 and 179 indicated the fission of the flavonoid B-ring. Quercetin-3-*O*-rutinoside (rutin) from petal and leaf extracts was identified based on its UV spectral characteristics, retention time and MS^n^ spectra and comparison with the corresponding reference standard. This flavonoid glycoside was not detected in the roots of the different *Primula* types. Quercetin-3-gentiobioside has been reported in *Primula veris* L. with yellow petals [37] and was also detected in the present study with a predominant signal at *m*/*z* 625. Moreover, a quercetin-trihexoside was determined, exhibiting a signal at *m*/*z* 787.

In addition to the phenolic compounds and anthocyanins, triterpenoid saponins were identified in various extracts. The occurrence of primula acid I was confirmed by its mass spectrometric signal at *m*/*z* 1104 and its retention time of 53.0 min, which was in accordance with the reference standard. Primula acid II was identified by its [M-H]^−^ ion at *m*/*z* 1236 and fragment ions at *m*/*z* 924 as well as *m*/*z* 465. Priverosaponin B-22-acetate with the [M-H]^−^ ion at *m*/*z* 1162 was confirmed by comparison with literature data [12,38].

## 5. Conclusions

In conclusion, the present investigations markedly broadens our knowledge of the phenolic constituents and triterpenoid saponins in *P. veris* variants with flower color mutations and complement the literature data of the therapeutically used *Primula* species.

As it is often unclear whether reported chemical diversity within a certain taxon is the result of natural chemotypes used for investigation or just a matter of application of different extraction and identification methods, it was our intention to establish and compare the metabolite profile of different plant organs of *P. veris* under standardized conditions. Hence, an extraction with methanol and add-on LC-MS analysis in the negative and positive ionization modes was employed and allowed the efficient and comparative phytochemical characterization of phenolic compounds and triterpenoid saponins in flowers, leaves and roots of the plants. Besides the organ-specific occurrence of the investigated metabolites in *P. veris*, we compared the chemical diversity in prominent secondary metabolites between the mutants and the wild type. In total, two flavonoids, six flavonoid glycosides, four methylated flavonoid glycosides, five anthocyanins and three triterpenoid saponins were identified in the present work. Except for the anthocyanins, significant differences in the saponin and flavonoid profiles of the mutants and the wild type were not observed, thus indicating that flower color mutation does not affect the potential pharmacological use of the respective plant species.

## Figures and Tables

**Table 1:** Spectroscopic data (UV, MS) and HPLC retention times (t_R_) of secondary metabolites from petal color variants of *Primula veris* L.

Compound	t_R_ (min)	λ_max_ (nm)	*m*/*z*	HPLC-ESI-MS/MS Fragments, *m*/*z*	Plant Variants									Reference
					Yellow Variant			Orange Variant			Red Variant			
					*p*	*l*	*r*	*p*	*l*	*r*	*p*	*l*	*r*	
**Flavonoids**			[M-H]^−^											
(+)-Catechin (**A**)	23.7	228, 280	289	245, 203, 186	**+**	−	−	**+**	−	−	**+**	−	−	st
Isorhamnetin	50.3	254, 368	315	300, 271, 151	−	−	−	**+**	−	−	**+**	−	−	st
**Flavonoid glycosides**			[M-H]^−^											
Quercetin-3-*O*-rutinoside (**H**)	32.1	256, 356	609	301, 179, 151	**+**	**+**	−	**+**	**+**	−	**+**	**+**	−	st
Quercetin-3-gentiobioside (**E**)	28.3	256, 354	625	463, 301, 179, 151	**+**	**+**	−	**+**	**+**	−	**+**	**+**	−	[15,16]
Quercetin-trihexoside (**C**)	25.5	256, 358	787	625, 463, 301, 179	**+**	**+**	−	**+**	**+**	−	**+**	**+**	−	[16]
Kaempferol-3-*O*-diglucoside-7-*O*-glucoside (**D**)	29.4	270, 350	771	609, 285, 257, 151	**+**	**+**	−	**+**	**+**	−	**+**	**+**	−	[17]
Kaempferol-3-*O*-rutinoside (**J**)	34.4	264, 350	593	447, 285, 257, 229	**+**	**+**	−	**+**	**+**	−	**+**	**+**	−	[15]
Kaempferol-3-*O*-galactoside-rhamnoside-7-*O*-rhamnoside (**F**)	30.1	254, 356	739	575, 429, 282, 255	**+**	**+**	−	**+**	**+**	−	**+**	**+**	−	[18]
**Methylated flavonoid glycosides**			[M-H]^−^											
Quercetin-methylether-rhamnoside-glucoside-rhamnoside (**G**)	31.5	254, 354	769	737, 623, 605, 315, 300, 271	**+**	**+**	−	**+**	**+**	−	**+**	**+**	−	[13]
Methyl-quercetin-dihexoside (**I**)	33.2	254, 356	639	477, 315, 300, 271	**+**	**−**	−	**+**	−	−	**+**	−	−	
Methyl-quercetin-rhamnoside-hexoside (**K**)	37.6	254, 356	623	477, 315, 300, 271	**+**	**+**	−	**+**	**+**	−	**+**	**+**	−	
Methyl-myricetin-trihexoside (**B**)	24.3	278, 342	817	655, 493, 331, 316, 271	**+**	−	−	**+**	−	−	**+**	−	−	
**Anthocyanins**			[M]^+^											
Cyanidin-3-*O*-glucoside (**M**)	18.1	280, 520	449	287, 231, 213	−	*	*	**+**	*	*	**+**	*	*	st
Cyanidin-hexoside (**L**)	17.3	280, 520	449	287, 231, 213	−	*	*	**+**	*	*	**+**	*	*	[19]
Peonidin-hexoside (**O1**)	19.6	280, 520	463	301, 286, 258	−	*	*	**+**	*	*	**+**	*	*	[19]
Peonidin-dihexoside (**O2**)	19.2	280, 520	625	301, 286, 258	−	*	*	**+**	*	*	**+**	*	*	[19]
Malvidin-hexoside (**P**)	20.2	260, 520	493	331, 315, 298, 281	−	*	*	**+**	*	*	**+**	*	*	[19]
**Triterpenoid saponins**			[M-H]^−^											
Primula acid I	53.1	−	1104	924, 465, 447, 246	**+**	**+**	**+**	**+**	**+**	**+**	**+**	**+**	**+**	st
Primula acid II	52.4	−	1236	924, 465	−	−	**+**	−	−	**+**	−	−	**+**	[12]
Priverosaponin B-22-acetate	52.1	−	1162	982, 465	−	−	**+**	−	−	**+**	−	−	**+**	[12]

*p*, petals; *l*, leaves; *r*, roots; **+**, detected; −, not detected; * not analyzed; (**A** to **P**), compounds are shown in Figure 2; st, reference standard used to verify retention time and/or UV spectrum and/or MS fragmentation pattern.
